# Warfarin Use During Fecal Occult Blood Testing: A Meta-Analysis

**DOI:** 10.4021/gr419w

**Published:** 2012-03-20

**Authors:** Imran Ashraf, Shafaq Paracha, Saif-ur-Rahman Paracha, Murtaza Arif, Abhishek Choudhary, Jonathan D. Godfrey, Robert E. Clark, Obai Abdullah, Michelle L. Matteson, Srinivas R. Puli, Jamal A. Ibdah, Ousama Dabbagh, Matthew L. Bechtold

**Affiliations:** a Five Hospital Drive, Department of Internal Medicine, University of Missouri - Columbia, Columbia, MO 65212, USA; b OSF MG Gastroenterology, 2805 N. Knoxville Avenue, Peoria, IL 61604, USA

**Keywords:** Fecal occult blood test, Warfarin, Colorectal cancer, Meta-analysis

## Abstract

**Background:**

Fecal occult blood testing (FOBT) is a widely used screening test for colorectal cancer (CRC). Given the limited data about the effects of warfarin on FOBT are inconclusive, current screening guidelines for CRC do not address whether warfarin should be discontinued before FOBT. Therefore, we conducted a meta-analysis to evaluate the influence of warfarin on the yield of FOBT.

**Methods:**

Multiple medical databases were searched (April 2011). Studies examining the use of warfarin versus no warfarin for FOBT were included. Meta-analysis for the effect of warfarin or no warfarin for FOBT was performed by calculating pooled estimates of colonoscopy findings and detection of neoplasia, any adenoma, advanced adenoma, or colon cancer by odds ratio (OR) with fixed and random effects model. RevMan 5.1 was utilized for statistical analysis.

**Results:**

Five studies (N = 11,244) met the inclusion criteria. No statistically significant difference was noted between FOBT with or without warfarin for colonoscopy findings (OR 0.88; 95% CI: 0.48 - 1.62, P = 0.67) or detection of neoplasia (OR 0.88; 95% CI: 0.58 - 1.35, P = 0.57), any adenoma (OR 1.08; 95% CI: 0.73 - 1.58, P = 0.71), advanced adenoma (OR 1.07; 95% CI: 0.69 - 1.65, P = 0.78), and colon cancer (OR 0.69; 95% CI: 0.38 - 1.23, P = 0.21).

**Conclusions:**

Among patients with positive FOBT, the yield of colonoscopy appears not to be altered by warfarin use.

## Introduction

Colorectal cancer is one of the leading causes of cancer related deaths in the western world with an approximate incidence of 150,000 new cases in the US and 30,000 new cases in the UK each year [1-4]. Randomized clinical trials have shown a significant reduction in CRC-related mortality by screening [5-7]. The American Cancer Society’s screening guidelines for CRC include yearly fecal occult blood testing (FOBT), or a flexible sigmoidoscopy every 5 years, or a colonoscopy every 10 years [8]. Therefore, being a less invasive test, FOBT is a widely used screening modality for CRC and has shown significant reduction in CRC-related mortality when coupled with subsequent colonoscopy [7, 9-11].

The use of FOBT for CRC detection relies on its ability to identify tumors which bleed, giving a positive result on FOBT [12]. However, false positives, for a variety of reasons, results in low specificity [13]. The use of anticoagulants at the time of obtaining FOBT has been considered a significant contributor to a false positive result [14, 15].

Warfarin is an anticoagulant which is amongst the top 200 medications prescribed in the U.S. and has been associated with overt gastrointestinal bleeding [16-19]. Current CRC screening guidelines and the hemoccult II test manufacturers do not specify whether warfarin should be stopped before FOBT [20-22]. Studies have shown conflicting results in the past regarding the yield of FOBT in patients on warfarin. Therefore, we conducted a meta-analysis to evaluate the evidence regarding the influence of warfarin on the yield of FOBT.

## Methods

### Data collection

Data collection was performed in three stages. First, a search was performed in MEDLINE, Cochrane Central Register of Controlled Trials and Database of Systematic Reviews, CINAHL, and PubMed in April 2011. Second, references of the retrieved articles and reviews were manually searched for any additional articles. Third, a manual search of abstracts submitted to the Digestive Disease Week (DDW) and the American College of Gastroenterology (ACG) national meetings was performed from 2003 - 2010. All articles were searched irrespective of language, publication status (articles or abstracts), or results. The keywords used for the search included “fecal occult blood test”, “warfarin”, “colorectal cancer”.

### Selection criteria

Independently, three authors (IA, SRP, and MLB) screened all of the articles and abstracts. Any disagreements in the data were resolved by a third party (AC). Articles were selected if they compared the findings on colonoscopies after positive FOBT among adult patients who were on warfarin or controls (no warfarin). Authors were contacted if data was incomplete or requiring clarification. We excluded studies if they did not compare warfarin to no warfarin populations.

### Statistical analysis

A meta-analysis for the effect of warfarin or no warfarin for FOBT was performed by calculating pooled estimates of colonoscopy findings and detection of neoplasia, any adenoma, advanced adenoma, or colon cancer using odds ratio (OR) by fixed and random effects models. The meta-analysis was performed in accordance to the guidelines published for meta-analysis of observational studies in epidemiology (MOOSE) [23]. Heterogeneity among studies was assessed by calculating I^2^ measure of inconsistency which was considered significant if P < 0.10 or I^2^ > 50%. If heterogeneity was statistically significant, a study elimination analysis was utilized to examine for heterogeneity when certain studies were excluded from the analysis. RevMan 5.1 was utilized for statistical analysis. Publication bias was assessed by funnel plots.

### Study quality assessment

Quality of cohort studies was assessed using the Newcastle-Ottawa quality assessment scale for cohort studies [24]. Briefly, this scale is based upon giving a star (★) for each of three quality parameters: Selection, comparability, and outcome. Stars may range from zero stars (very poor quality cohort study) to nine stars (very strong quality cohort study) [24]. Studies with 7 stars or greater are considered high-quality studies.

## Results

### Literature search

**Figure 1:**
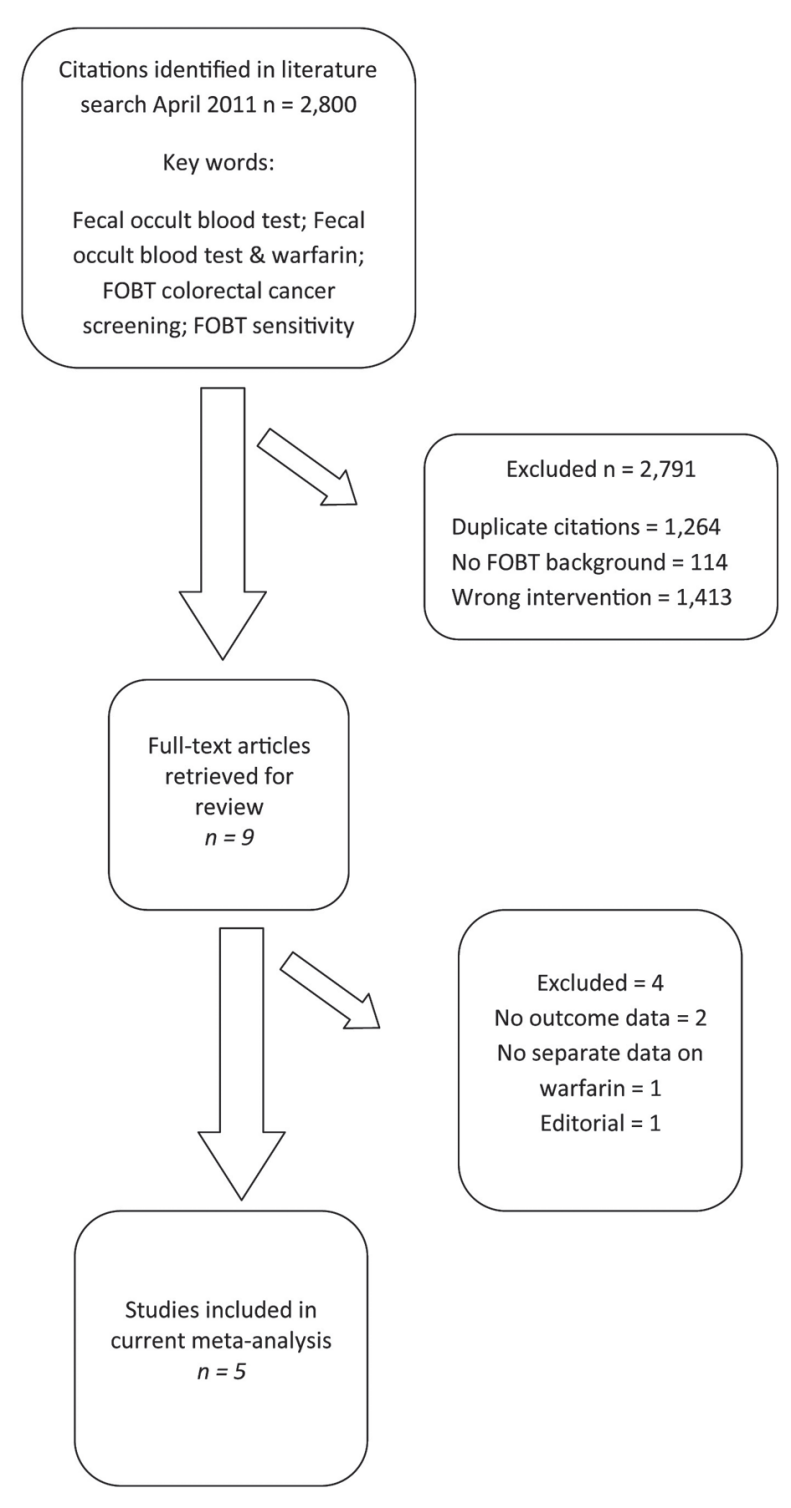
Selection of studies for inclusion in the meta-analysis of warfarin use during fecal occult blood testing.

We identified 2,800 articles and abstracts through the electronic database search (Fig. 1). Of the 2,800 citations identified, we excluded 2,791 after screening the titles and abstracts. Of the remaining, nine were examined by full-text review. Of these nine articles, four were excluded (no outcome data = 2, outcome unrelated = 1, and no FOBT = 1). We included five published articles in our current meta-analysis [14, 25-28].

### Study characteristics

Of the five included trials (N = 11,244), one was a prospective cohort [25] and the others were retrospective cohort studies [14, 26-28]. The studies were performed at various locations in the United States, Israel, and Italy between 2005 and 2010. All the studies included patients with a positive FOBT with subsequent colonoscopy. Studies were of adequate quality as assessed by the Newcastle-Ottawa quality assessment scale (Table 1)

**Table 1:** Characteristics of Studies Included in Meta-Analysis

Author	Study	Blinded	Location	FOBT	Patients (n)	Study Quality ★
Bini et al - 2005	Cohort	None	United States	Hemoccult II	420	★★★★★★★★★
Iles-Shih et al - 2010	Cohort	None	United States	Hemoccult II	9637	★★★★★★★★★
Kershenbaum et al - 2010	Cohort	None	Israel	Hemoccult Sensa	425	★★★★★★★★
Sawhney et al - 2010	Cohort	None	United States	Hemoccult II or equivalent	603	★★★★★★★★
Mandelli et al - 2010	Cohort	None	Italy	iFOBT	159	★★★★★★★★

★ Stars based upon Newcastle-Ottawa quality assessment scale for cohort studies (0 stars = poor, 9 stars = excellent)

### Analysis

**Figure 2:**
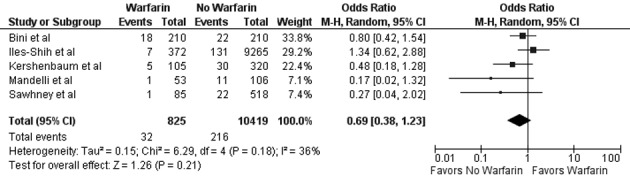
Forest plot showing no statistically significant effect on colorectal cancer detection for FOBT obtained with warfarin versus without warfarin.

**Figure 3:**
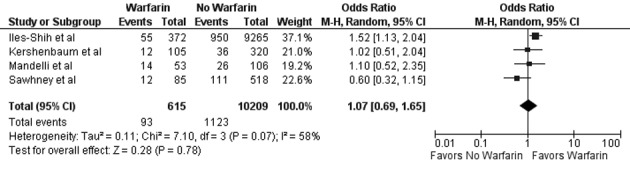
Forest plot showing no statistically significant effect on advanced adenoma detection for FOBT obtained with warfarin versus without warfarin.

**Figure 4:**
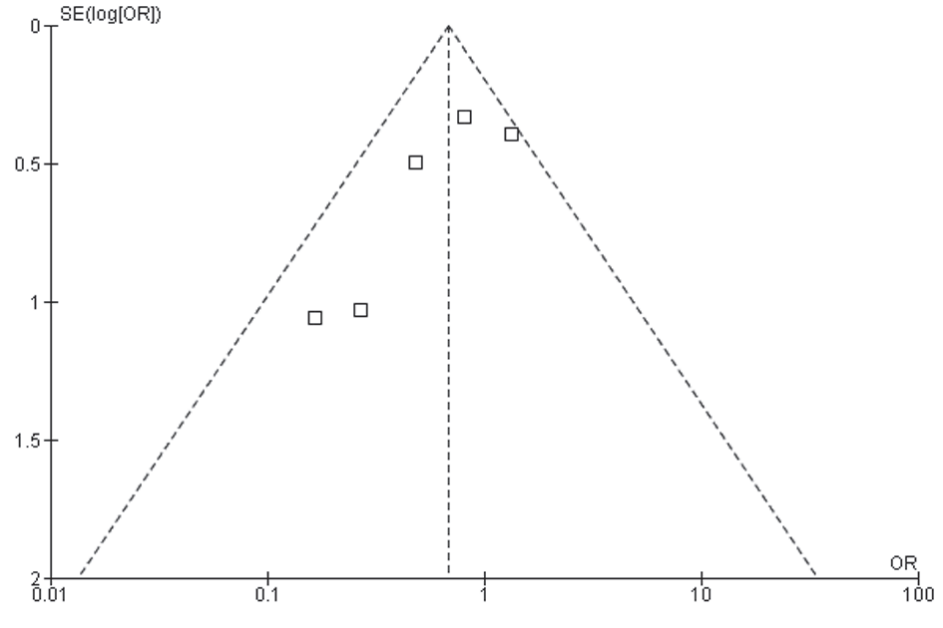
Measure of publication bias using a funnel plot.

In our meta-analysis of observational studies, no statistically significant differences were noted between FOBT with or without warfarin for the primary outcomes of colon cancer (OR 0.69; 95% CI: 0.38 - 1.23, P = 0.21) and advanced adenoma (OR 1.07; 95% CI: 0.69 - 1.65, P = 0.78) (Fig. 2 and 3). For the secondary outcomes, no statistically significant differences were noted between FOBT with or without warfarin for colonoscopy findings (OR 0.88; 95% CI: 0.48 - 1.62, P = 0.67) and detection of neoplasia (OR 0.88; 95% CI: 0.58 - 1.35, P = 0.57) or any adenoma (OR 1.08; 95% CI: 0.73 - 1.58, P = 0.71) (Table 2). No publication bias was identified (Fig. 4). Statistically significant heterogeneity was noted for three outcomes (colonoscopic findings, any adenoma, and advanced adenoma). A study elimination analysis was performed for colonoscopic findings (OR 1.18; 95% CI: 0.79 - 1.76, P = 0.41; I^2^ = 34%, P = 0.22) and detection of neoplasia (OR 0.86; 95% CI: 0.65 - 1.14, P = 0.30; I^2^ = 48%, P = 0.14) or advanced adenoma (OR 0.85; 95% CI: 0.57 - 1.27, P = 0.43; I^2^ = 0%, P = 0.40) with similar findings.

**Table 2:** Summary of the Analyses for FOBT Obtained With Warfarin Versus Without Warfarin for Colonoscopy Findings and Detection of Neoplasia or Any Adenoma

Outcome	Odds Ratio	95% Confidence Interval	P-Value	I^2^
Colonoscopic Findings	0.88	0.48 - 1.62	0.67	81%*
Neoplasia	0.88	0.58 - 1.35	0.57	76%*
Any Adenoma	1.08	0.73 - 1.58	0.71	23%

* Random effects model and an elimination analysis was performed given statistically significant heterogeneity.

## Discussion

Colorectal cancer is a prevalent disease that has a significant impact on individuals as well as healthcare costs. Many screening modalities are available for CRC screening, with FOBT being the least invasive. As with all screening tests, FOBT has its limitations, mostly with false positive results which may lead to unnecessary subsequent colonoscopies. Some reports in the literature have suggested that concomitant use of anticoagulation, such as warfarin, with FOBT may influence the results, leading to more false positives. However, the literature has not been conclusive on this subject, showing conflicting results.

Kewenter et al considered FOBT not appropriate for CRC-screening in patients on anticoagulation because of its too low sensitivity and positive predictive value in this setting [29]. However, their study lacked a control group and a colonoscopy was not performed in all the patients with a positive FOBT. Blackshear et al showed an increased level of mean fecal hemoglobin in patients taking warfarin but did not investigate it further with a colonoscopy [30]. Clarke et al conducted a prospective study that favored stopping warfarin before FOBT based upon a statistically significant reduction in the positive predictive value of neoplastic lesions; however, their reported results were for all the antithrombotics and warfarin [15]. Similarly, Sawhney et al performed a retrospective analysis which favored stopping warfarin before FOBT because of a lower positive predictive value for advanced neoplasia for persons taking warfarin [14]. Despite these studies suggesting the use of warfarin may influence FOBT results, other studies have been performed with differing findings.

Greenberg et al and Prichard et al in two separate studies did not show any increase in GI blood loss associated with warfarin [31, 32]. Bini et al found no significant difference in the positive predictive value of FOBT between the two groups, thereby supporting continued use of warfarin during FOBT [25]. Similarly, Levi et al suggested that warfarin and other antithrombotics may increase the sensitivity of FOBT [33]. More recently, Iles-Shih et al and Kershenbaum et al also favored continuing warfarin for FOBT [26, 27]. Given these conflicting results in the literature and the importance of assessing the affects of warfarin on FOBT, we conducted this meta-analysis.

In this meta-analysis, we document that FOBT is not influenced by continuation of warfarin therapy. We found no statistically significant differences between FOBT results for patients taking warfarin or not taking warfarin for findings on colonoscopy and detection of neoplasia, including any adenoma, advanced adenoma, or colon cancer.

The strengths of this meta-analysis are numerous. First, a comprehensive article and abstract screening process with an extensive three-stage search technique was utilized to maximize article recognition. Second, a large number of patients in various populations were examined. Third, all studies evaluated the primary two outcomes (advanced adenomas and colon cancer). Fourth, this meta-analysis included positive and negative high-quality observational studies per Newcastle-Ottawa scale. Fifth, no publication bias was noted. Finally, this represents the first meta-analysis to-date assessing the yield of FOBT in patients on warfarin with the potential to alter everyday clinical practice, especially in the primary care setting. On the other hand, our meta-analysis also had a few limitations. First, the study quality was not ideal given lack of randomized controlled trials. However, it must be noted that no randomized controlled trials have been performed on this particular subject. Also, given the use of observational cohort studies, the meta-analysis was performed using the MOOSE guidelines specifically designed for observational studies and the quality of the studies were assessed using the Newcastle-Ottawa scale. Second, in the Kershenbaum et al study, 24% patients did not have a follow-up colonoscopy [27]. However, patients were equally distributed between the two groups which minimized its effect. Third, different FOBT mechanisms were utilized in the study, consistent with usage around the world. Most studies analyzed Hemoccult II or Hemoccult Sensa FOBT except Mandelli et al who analyzed the immunochemical FOBT (iFOBT). Given this meta-analysis focused on the effect of warfarin on FOBT and since many formulations of FOBT are currently available and used, we analyzed the overall effect. Further studies need to be performed using iFOBT for future analysis. Finally, heterogeneity was identified in one primary outcome (advanced adenoma) and two secondary outcomes (colonoscopic findings and any adenoma). To minimize the effect, a random effects model was utilized. Also, an elimination analysis was performed which demonstrated similar results without heterogeneity. Therefore, heterogeneity did not seem to influence the overall results.

In conclusion, the use of warfarin does not seem to affect FOBT. Therefore, based upon this information, patients on chronic anticoagulation with warfarin do not require cessation of the medication for adequate FOBT to screen for colorectal cancer.
